# Incidence of Acute Chest Syndrome in Children With Sickle Cell Disease Following Implementation of the 13-Valent Pneumococcal Conjugate Vaccine in France

**DOI:** 10.1001/jamanetworkopen.2022.25141

**Published:** 2022-08-02

**Authors:** Zein Assad, Morgane Michel, Zaba Valtuille, Andrea Lazzati, Priscilla Boizeau, Fouad Madhi, Jean Gaschignard, Luu-Ly Pham, Marion Caseris, Robert Cohen, Florentia Kaguelidou, Emmanuelle Varon, Corinne Alberti, Albert Faye, François Angoulvant, Bérengère Koehl, Naïm Ouldali

**Affiliations:** 1 Department of General Pediatrics, Pediatric Infectious Disease and Internal Medicine, Robert Debré University Hospital, Assistance Publique-Hôpitaux de Paris, Paris, France; 2 Clinical Epidemiology Unit, Robert Debré University Hospital, Assistance Publique-Hôpitaux de Paris, Paris, France; 3 Université Caen-Normandie, Caen, France; 4 Unité de Recherche Clinique en Économie de la Santé, Hôtel-Dieu, Assistance Publique-Hôpitaux de Paris, Paris, France; 5 ECEVE (Epidémiologie Clinique et Evaluation Economique Appliquées aux Populations Vulnérables), Institut national de la santé et de la recherche médicale, Unité mixte de recherche 1123, Paris University, Paris, France; 6 Institut national de la santé et de la recherche médicale, Centre d'Investigation Clinique 1426, Robert Debré University Hospital, Assistance Publique-Hôpitaux de Paris, Paris, France; 7 Department of General Surgery, Centre Hospitalier Intercommunal de Créteil, Créteil, France; 8 Department of General Pediatrics, Centre Hospitalier Intercommunal de Créteil, Créteil, France; 9 Department of General Pediatrics, Groupe Hospitalier Nord Essonne, Longjumeaux, France; 10 IAME (Infection, Antimicrobials, Modelling, Evolution), Institut national de la santé et de la recherche médicale, Unité mixte de recherche 1137, Paris University, Paris, France; 11 Department of General Pediatrics, Jean Verdier University Hospital, Assistance Publique-Hôpitaux de Paris, Bondy, France; 12 Association Clinique et Thérapeutique Infantile du Val-de-Marne, St Maur-des-Fossés, France; 13 Centre Hospitalier Intercommunal, Research Centre, Université Paris Est, IMRB-GRC GEMINI, Créteil, France; 14 National Reference Center for Pneumococci, Laboratoire de Microbiologie, Assistance Publique-Hôpitaux de Paris, Hôpital Européen Georges-Pompidou, Paris, France; 15 Centre de Recherche des Cordeliers, Institut national de la santé et de la recherche médicale (Unité mixte de recherche S1138), Sorbonne Université, Université de Paris, Paris, France; 16 Department of Child Hematology, Reference Center for Sickle-Cell Disease Robert Debré University Hospital, Assistance Publique-Hôpitaux de Paris, Paris, France; 17 Integrated Biology of Red Blood Cells, Institut national de la santé et de la recherche médicale, Unité mixte de recherche S1134, Paris University, Paris, France

## Abstract

**Importance:**

Acute chest syndrome (ACS) is one of the leading acute severe complications of sickle-cell disease (SCD). Although *Streptococcus pneumoniae* (*S pneumoniae*) is highly prevalent in children with SCD, its precise role in ACS is unclear. The efficacy of 13-valent pneumococcal conjugate vaccine (PCV13) implementation on ACS is still unknown.

**Objective:**

To assess the association of PCV13 implementation in the general pediatric population with the incidence of ACS in children with SCD.

**Design, Setting, and Participants:**

This cohort study used an interrupted time-series analysis of patient records from a national hospital-based French surveillance system. All children younger than 18 years with SCD (based on the *International Statistical Classification of Diseases and Related Health Problems, Tenth Revision* definition) hospitalized in France between January 2007 and December 2019 were included.

**Exposures:**

PCV13 implementation.

**Main Outcomes and Measures:**

Monthly incidence of ACS per 1000 children with SCD over time as analyzed by segmented linear regression with autoregressive error; monthly incidence of hospitalization for vaso-occlusive crisis, asthma crisis, and acute pyelonephritis per 1000 children with SCD over the same period as the control outcomes.

**Results:**

Among the 107 694 hospitalizations of children with SCD, 4007 episodes of ACS were included (median [IQR] age, 8 [4-12] years; 2228 [55.6%] boys). PCV13 implementation in 2010 was followed by a significant decrease in the incidence of ACS (−0.9% per month; 95% CI, −1.4% to −0.4%; *P* < .001), with an estimated cumulative change of −41.8% (95% CI, −70.8% to −12.7%) by 2019. Sensitivity analyses yielded the same results, including the incidence of ACS adjusted for that of vaso-occlusive crisis over time. The results were similar among different age groups. By contrast, no change was found for the 3 control outcomes over the study period.

**Conclusions and Relevance:**

PCV13 implementation was associated with an important reduction in the incidence of ACS in children with SCD. This vaccine benefit provides new evidence of the key role of *S pneumoniae* in ACS and should be considered when estimating outcomes associated with current PCVs and the potential benefit of next-generation PCVs in children.

## Key Points

**Question:**

Is the implementation of 13-valent pneumococcal conjugate vaccine (PCV13) in the general pediatric population associated with a change in the incidence of acute chest syndrome (ACS) in children with sickle cell disease?

**Findings:**

This cohort study including 107 694 hospitalizations used interrupted time series analysis of a prospective national surveillance cohort from 2007 to 2019. There was a significant decrease in the incidence of ACS after PCV13 implementation in 2010.

**Meaning:**

These results provide new evidence of the key role of *Streptococcus pneumoniae* in ACS and should be considered when estimating the public health benefit of current and next-generation pneumococcal conjugate vaccines in children.

## Teaser

This cohort study of children with sickle cell disease (SCD) in a French hospital system examines the association of 13-valent pneumococcal conjugate vaccine implementation with incidence of acute chest syndrome (ACS).

## Introduction

Sickle-cell disease (SCD) is one of the most common hemoglobinopathies worldwide, with approximately 300 000 new cases each year.^1^ Acute chest syndrome (ACS) is a specific severe complication of SCD, defined as the combination of chest pain, dyspnea, fever, and pulmonary infiltrates on chest x-ray.^2^ More than half of patients with homozygous SCD experience at least 1 episode of ACS in the first decade of life,^3^ 10% require mechanical ventilation, and overall mortality is approximately 3% in children.^4^

ACS shows a complex physiopathology with multiple suggested causes.^3^ Vichinsky et al^4^ estimated that infection may be involved in 25% of ACS episodes in children. Among the main infectious organisms identified in the study, *Streptococcus pneumoniae* (*S pneumoniae*) was estimated to be responsible for 4.5% of ACS. However, bacterial involvement in ACS is highly difficult to confirm because of limited reliable microbiological documentation.^4^ Therefore, the precise involvement of *S pneumoniae* in ACS is yet to be clarified.

*S pneumoniae* infections represent a major source of morbidity and mortality worldwide, with at least 294 000 deaths in children under 5 years of age in 2015.^5^ Children with SCD have a higher propensity to be infected by *S pneumoniae*, with a 100-fold higher rate of *S pneumoniae* infection than non-SCD children.^6^ The 7-valent pneumococcal conjugate vaccine (PCV7) was implemented in the 2000s, and had a strong impact on invasive pneumococcal disease (IPD) in children with SCD.^7^ However, outcomes for non-IPD were reported to be more modest, and the emergence of non-PCV7 serotypes eroded the benefit both for children with SCD and in the general pediatric population.^8,9^ To fight serotype replacement, the 13-valent pneumococcal conjugate vaccine (PCV13) has been used in the general population since 2010 in many countries, including France, and has been shown to have a significant reduction in IPD^10,11^ and non-IPD, including lower respiratory-tract infections.^12^ However, the public health outcomes of PCV13 implementation concerning ACS are still unknown. In this context, we aimed to assess the association of PCV13 implementation with incidence of ACS in children with SCD.

## Methods

### Study Design

This cohort study used a quasi-experimental, population-based, interrupted time series (ITS) analysis of patient data from a hospital-based French national surveillance system over 13 years (January 2007 to December 2019). The Strengthening the Reporting of Observational Studies in Epidemiology (STROBE) reporting guidelines have been followed to report this study. Access to the database was requested from and approved by the National Commission on Information and Liberty. As it is part of an ongoing continuous mission of public health, and used anonymous aggregated data for public health purposes, our study did not require ethical committee approval or written informed consent based on guidelines in French law from the 2021 National Data Protection Act.^13^

### Study Data and Settings

The data were extracted from the French Medicalization of Information Systems Program (PMSI), which is an exhaustive national medico-administrative database that includes all inpatients admitted to any public or private hospital in France. This coding system was initially established to analyze hospital medical activity described in a program and compiles discharge summaries for all admissions. The information recorded are anonymous and include demographic data, comorbidities, diagnoses related to the hospitalization, organ support, and death. Diagnoses identified during the hospital stay are recorded according to the *International Statistical Classification of Diseases and Related Health Problems, Tenth Revision *(*ICD-10*) and following a national guideline for the coding of each diagnosis (Technical Agency for Hospital Information [ATIH]^14^).

### Inclusion Criteria and Data Extracted

We included all children aged 18 years or younger with SCD hospitalized for ACS between January 2007 and December 2019. Based on the national ATIH guidelines, ACS was defined as the combination of the *ICD-10* discharge diagnosis code for SCD with crisis (D57.0 or D57.2) and 1 of several *ICD-10* codes for respiratory impairment (J96.0, J18.9, I26.9).

All children aged younger than 18 years with SCD hospitalized for pneumonia, other lower respiratory tract infections (LRTIs), asthma crises, acute pyelonephritis, and vaso-occlusive crises (VOC) were also recorded. Based on the national ATIH guidelines, pneumonia was defined as the combination of SCD without crisis (D75.1) and 1 of the following *ICD-10* codes for pneumonia: J13, J15.9, J18, J18.0, J18.1, J18.9, J85.1, J90. The details of ACS, pneumonia, other LRTIs, asthma crises, acute pyelonephritis, and VOC *ICD-10* codes are presented in eTable 1 in the Supplement. The data were analyzed at the hospitalization level; an individual child could contribute more than 1 hospitalization to the analysis. The following data were extracted for each inpatient stay: age, gender, date and duration of hospital stay, ventilatory support, transfer to an intensive care unit (ICU), and hospital death. Race and ethnicity data were not included in the PMSI database and have not been considered to affect outcomes in previous studies on SCD.

### National Immunization Program

PCV7 was licensed in France in 2001 and surpassed 80% vaccine coverage in 2008.^15^ In June 2010, it was recommended to shift from PCV7 to PCV13 for all children younger than 2 years of age (2 primary doses at ages 2 and 4 months and a booster at age 11 months) without catch up. For children with SCD, the immunization schedule consisted of 3 doses at ages 2, 3, and 4 months, followed by a booster dose at 12 months. For children with SCD who had started immunizations with PCV7, a catch-up and an additional dose of PCV13 was recommended.^16^ Since 2011, PCV13 coverage at age 24 months has been greater than 90% in the general population.^15^

We defined 2 periods according to PCV13 implementation in France: the pre-PCV13 period, from January 2007 to May 2010, and the PCV13 period, from June 2011 to December 2019. We used a transition period between the 2 periods because of the usual delay between the changes in national vaccination policies and their full implementation.^10,17^ In addition to PCV, the 23-valent-pneumococcal polysaccharide vaccine (PPSV23), marketed in France since 1981, was recommended for children 2 years or older and adults with a higher risk of IPD, with a booster dose every 5 years.^18^

### Outcomes

The main outcome was the monthly incidence of ACS per 1000 children with SCD in France. Secondary outcomes were the monthly incidence of ACS among different age groups (0 to 5 years, 6 to 10 years, 11 to 14 years, 15 to 17 years), the proportion of ventilatory support (ie, noninvasive ventilation or invasive ventilation), and transfer to an ICU among ACS episodes over time. Furthermore, because of the large potential overlap between ACS and pneumonia in children with SCD, several studies suggested to combine these 2 entities to avoid misclassification between them.^19,20^ To take this into account, we conducted an analysis assessing the association of PCV13 with the incidence of combined ACS and pneumonia per 1000 children with SCD in France.

We calculated the incidence per 1000 children with SCD using the monthly number of ACS episodes as numerator and the number of children with SCD under 18 years of age living in France as denominator for each year of the study (eFigures 1 and 2 in the Supplement). This information was provided by the National Health Insurance Scheme database.^21^ As recommended in previous studies about ITS methodology,^22,23^ we assessed the risk of bias due to potential hidden cointerventions in ITS analyses by analyzing 3 control outcomes over the same period that would not be expected to be influenced by PCV13 implementation: changes in the monthly incidence of VOC, asthma crises, and acute pyelonephritis per 1000 children with SCD.

### Statistical Analysis

We analyzed the main outcome by segmented linear regression with autoregressive error.^22^ Seasonality was accounted for using an additive model. We used an autoregressive–moving-average term to account for the remaining autocorrelation. The time unit chosen was 1 month, to provide sufficient number of events per time unit and enough statistical power.^24^

We hypothesized that the intervention would have a progressive effect.^25^ Thus, the intervention assessment involved a dummy variable in the model for each period (pre-PCV13, PCV13), estimating the trend before the intervention and the change in slope following PCV13 implementation. The estimated cumulative reduction in incidence was expressed as the percentage change between the incidence fitted by the model and the estimated counterfactual incidence, which was calculated for each time point of the postintervention period. The validity of the segmented regression model was assessed by visual inspection of the correlograms (autocorrelation and partial autocorrelation functions) and residuals analysis. We checked whether the residuals of the models were normally distributed and showed constant variance over time.^23^

Seven sensitivity analyses were performed to assess the robustness of the study findings: (1) a quasi-Poisson regression model accounting for seasonality using harmonic terms (sines and cosines), (2) a segmented linear regression adjusted for the monthly incidence of VOC over the same period to explore the possibility that potential changes observed in the incidence of ACS may be related to changes in VOC incidence, (3) a segmented linear regression model with ACS incidence defined as the combination of both ACS and pneumonia episodes, (4) a segmented linear regression model with *ICD-10* codes for pneumonia (J18.9) and for sickle cell and hemoglobin-C disease (D57.2) excluded from the ACS definition to explore the potential overlap between ACS and pneumonia, (5) a segmented linear regression analysis without a transitional period, (6) a segmented linear regression model with seasonality accounted for by harmonic terms (sines and cosines) with 12-month periods, and (7) a segmented linear regression model including harmonic terms with 3-, 6-, and 12-month periods to explore potential nonyearly seasonal patterns.

A 2-sided *P* value <.05 was considered significant. The data were extracted from the PMSI database using SAS version 9.4 (SAS Institute) and statistical analyses were performed using R version 4.1.1 (R Project for Statistical Computing).

## Results

### Characteristics of ACS and Other Acute Respiratory Diseases

Between January 2007 and December 2019, 107 694 hospitalizations of children with SCD were included (median [IQR] age, 9 [4-13] years; 56 264 [52.2%] boys). ACS accounted for 4007 (3.7%) cases, pneumonia 1789 (1.7%), other LRTIs 1153 (1.1%), asthma crises 845 (0.8%), acute pyelonephritis 889 (0.8%), and VOC 69 920 (64.9%).

ACS showed different characteristics than hospitalizations for other acute respiratory diseases among children with SCD (Table 1). First, the median age of patients with ACS was older (8 [4-12] years vs 4 [2-7] years for pneumonia, 1 [0.6-3] year for other LRTIs, and 5 [2-10] years for asthma crises). Second, the seasonal pattern also differed, with a 21.2% decrease in summer vs winter compared with a 41.9% decrease for pneumonia, an 80.0% decrease for other LRTIs, and a 13.8% decrease for asthma crises. Third, patients with ACS had more severe outcomes, with a higher ICU admission rate (868 of 4007 patients [21.7%] vs 98 of 1789 [5.1%] for pneumonia, 17 of 1153 [1.5%] for other LRTIs, and 20 of 845 [2.4%] for asthma crises) and a greater need for ventilatory support (946 of 4007 patients [23.6%] vs 114 of 1789 [6.4%] for pneumonia, 20 of 1153 [1.7%] for other LRTI, and 20 of 845 [2.4%] for asthma crises). The proportions of the *ICD-10* code combinations to define ACS did not change during the study period and among the age groups (eTable 2 in the Supplement).

**Table 1.** Baseline Characteristics of Hospitalizations of Children With Sickle Cell Disease by Discharge Diagnosis, January 2007 to December 2019

Characteristics	Children, No. (%)^a^							
	ACS	Asthma crisis	Pneumonia	Other LRTIs^b^	Acute pyelonephritis	VOC	Other diagnosis^c^	Total cases
No. of cases	4007 (3.7)	845 (0.8)	1789 (1.7)	1153 (1.1)	889 (0.8)	69 920 (64.9)	29 091 (27.0)	107 694
Age, median (IQR), y	8 (4-12)	5 (2-10)	4 (2-7)	1 (0.6-3)	1 (0.9-7)	9 (4-13)	5 (2-11)	9 (4-13)
Age groups, y								
0-5	1367 (34.1)	472 (55.9)	1181 (66.0)	989 (85.8)	631 (71.0)	21 981 (31.4)	14 392 (49.5)	41 013 (38.1)
6-10	1235 (30.8)	183 (21.7)	389 (21.7)	108 (9.4)	110 (12.4)	19 381 (27.7)	6701 (23.0)	28 107 (26.1)
11-14	823 (20.5)	116 (13.7)	130 (7.3)	35 (3.0)	87 (9.8)	16 276 (23.3)	4444 (15.3)	21 911 (20.3)
15-17	581 (14.5)	74 (8.8)	89 (5.0)	21 (1.8)	60 (6.7)	12 185 (17.4)	3146 (10.8)	16 156 (15.0)
Sex								
Boys	2228 (55.6)	525 (62.1)	907 (50.7)	670 (58.1)	381 (42.9)	35 499 (50.8)	16 054 (55.2)	56 264 (52.2)
Girls	1779 (44.4)	320 (37.9)	882 (49.3)	483 (41.9)	508 (57.1)	34 421 (49.2)	13 037 (44.8)	51 430 (47.8)
Seasonal pattern, % (SD)^d^	−21.2 (8.8)	−13.8 (47.5)	−41.9 (19.4)	−80.0 (8.8)	−6.0 (37.9)	−4.7 (6.0)	−3.6 (6.6)	−7.5 (4.2)
Outcome								
Duration of stay, median (IQR), d	7 (4-9)	3 (2-4)	4 (3-7)	3 (2-4)	4 (3-6)	3 (1-5)	2 (0-3)	3 (1-5)
ICU admission	868 (21.7)	20 (2.4)	98 (5.1)	17 (1.5)	9 (1.0)	878 (1.3)	586 (2.0)	2476 (2.3)
Ventilatory support^e^	946 (23.6)	20 (2.4)	114 (6.4)	20 (1.7)	10 (1.1)	1113 (1.6)	751 (2.6)	2974 (2.8)
Hospital death	18 (0.4)	0	2 (0.1)	0	0	49 (0.1)	48 (0.2)	117 (0.1)

Abbreviations: ACS, acute chest syndrome; ICU, intensive care unit; LRTIs, lower respiratory tract infections; VOC, vaso-occlusive crisis.

^a^ Missing values for age groups were 1 child (0.02%) for ACS, 1 (0.1%) for acute pyelonephritis, 97 (0.1%) for VOC, 408 (1.4%) for other diagnosis, and 507 (0.5%) for total cases; for duration of stay, data were missing for 2 children (0.001%) for pneumonia, 99 (0.001%) for VOC, 268 (0.009%) for other diagnosis, and 369 (0.003%) for total cases.

^b^ Other LRTIs include acute bronchiolitis, acute bronchitis, and other viral LRTIs.

^c^ Other diagnosis includes nonspecific fever, renal dialysis, upper respiratory tract infection, gastroenteritis, splenic sequestration, hypersplenism, and blood transfusion.

^d^ Seasonal pattern represents the percentage decrease in summer vs winter seasons.

^e^ Ventilatory support includes invasive and noninvasive ventilation.

### Association of PCV13 Implementation With the Incidence of ACS

**Figure 1.**
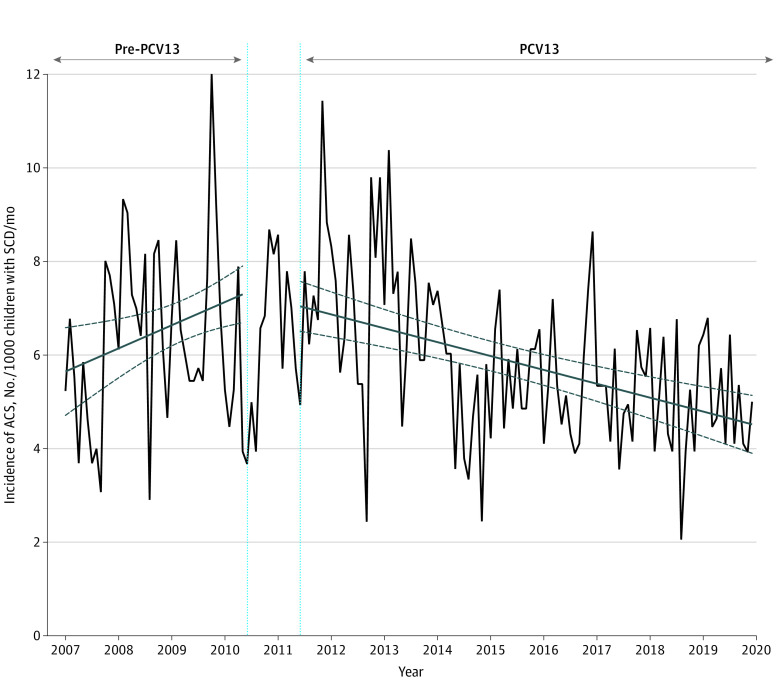
Association of 13-Valent Pneumococcal Conjugate Vaccine (PCV13) Implementation With the Monthly Incidence of Acute Chest Syndrome (ACS) per 1000 Children With Sickle Cell Disease (SCD) in France (N = 4007) The dark blue lines indicating the slope were estimated using the segmented linear regression model; dashed dark blue lines show the 95% CI. The dotted light blue vertical lines indicate the transition period during which PCV13 was implemented. The pre-PCV13 period was from January 2007 to May 2010; the transitional period, from June 2010 to May 2011; the PCV13 period, from June 2011 to December 2019.

The incidence of ACS estimated by the interrupted time-series model was 7.3 cases per 1000 children with SCD in May 2010 and 4.5 cases per 1000 children in December 2019. The implementation of PCV13 in June 2010 was followed by a significant decrease in ACS episodes during the 8 postintervention years (change in slope, −0.9% per month; 95% CI, −1.4% to −0.4%; *P* < .001) (Table 2, Figure 1; eTable 3 in the Supplement). The estimated cumulative reduction of the incidence of ACS following PCV13 implementation by the end of the study was −41.8% (95% CI, −70.8% to −12.7%) (Table 2). The quality assessment of the final model was satisfactory (eFigure 3 in the Supplement). All sensitivity analyses provided similar results, including the incidence of ACS adjusted for the monthly incidence of VOC over time, and the models exploring the potential overlap between ACS and pneumonia (Table 2; eFigures 4, 5, 6, and 7 and eTable 4 in the Supplement).

**Table 2.** Association of 13-Valent Pneumococcal Conjugate Vaccine (PCV13) Implementation With the Monthly Incidence of Acute Chest Syndrome (ACS) in Children With Sickle Cell Disease (SCD)

Outcome	Change in slope per mo, % (95% CI)	*P* value for slope change	Estimated cumulative change by the end of the study, % (95% CI)
Monthly incidence of ACS per 1000 children with SCD^a^^,^^b^	−0.9 (−1.4 to −0.4)	<.001	−41.8 (−70.8 to −12.7)
Sensitivity analyses			
Quasi-Poisson regression model	−0.9 (−1.5 to −0.4)	<.001	−41.2 (−81.0 to −1.4)
Segmented linear regression model with trigonometric function (12 m)	−0.9 (−1.3 to −0.4)	<.001	−35.1 (−62.0 to −8.1)
Segmented linear regression model with trigonometric function (3-6-12 m)	−0.8 (−1.2 to −0.3)	<.001	−35.0 (−64.9 to −5.0)
Model adjusted for the monthly incidence of VOC^b^	−0.9 (−1.4 to −0.4)	.001	−40.3 (−70.4 to −10.1)
Model with combined ACS and pneumonia^b^	−0.5 (−0.9 to −0.1)	.03	−27.5 (−57.1 to 2.0)
Model with J18.9 and D57.2 excluded from ACS definition^b^	−1.4 (−2.0 to −0.8)	<.001	−52.9 (−82.6 to −23.2)
Segmented linear regression model excluding transition period	−0.8 (−1.3 to −0.3)	.002	−35.0 (−62.1 to −7.8)
Monthly incidence of ACS by age group, y^a^^,^^b^			
0-5	−0.9 (−1.6 to −0.1)	.03	−41.8 (−88.1 to 4.5)
6-10	−0.9 (−1.6 to −0.2)	.01	−43.1 (−82.9 to −3.4)
11-14	−0.7 (−1.9 to 0.4)	.19	−34.3 (−97.3 to 28.7)
15-17	−1.4 (−2.3 to −0.5)	.003	−52.8 (−95.7 to −9.9)
Secondary outcomes			
Proportion of ventilatory support among ACS^c^^,^^b^	−1.1 (−2.3 to 0.1)	.07	−33.7 (−78.7 to 11.4)
Proportion of ICU transfer among ACS^b^	0.9 (−0.4 to 2.1)	.18	52.7 (−41.5 to 147.0)
Control outcomes			
Monthly incidence of VOC per 1000 children with SCD^a^^,^^b^	−0.1 (−0.3 to 0.1)	.30	- 5.1 (−17.1 to 6.9)
Monthly incidence of asthma crises per 1000 children with SCD^a^^,^^b^	0.01 (−1.1 to 1.1)	.98	0.8 (−74.7 to 76.2)
Monthly incidence of acute pyelonephritis per 1000 children with SCD^a^^,^^b^	0.5 (−0.5 to 1.5)	.30	39.4 (−53.0 to 131.8)

Abbreviations: ICU, intensive care unit; VOC, vaso-occlusive crisis.

^a^ Monthly incidence expressed as the number of cases per 1000 children with SCD.

^b^ Analysis by segmented linear regression.

^c^ Ventilatory support includes invasive and noninvasive ventilation.

### Incidence of ACS by Age Group and Proportion of Ventilatory Support and Transfer to ICU Over Time

**Figure 2.**
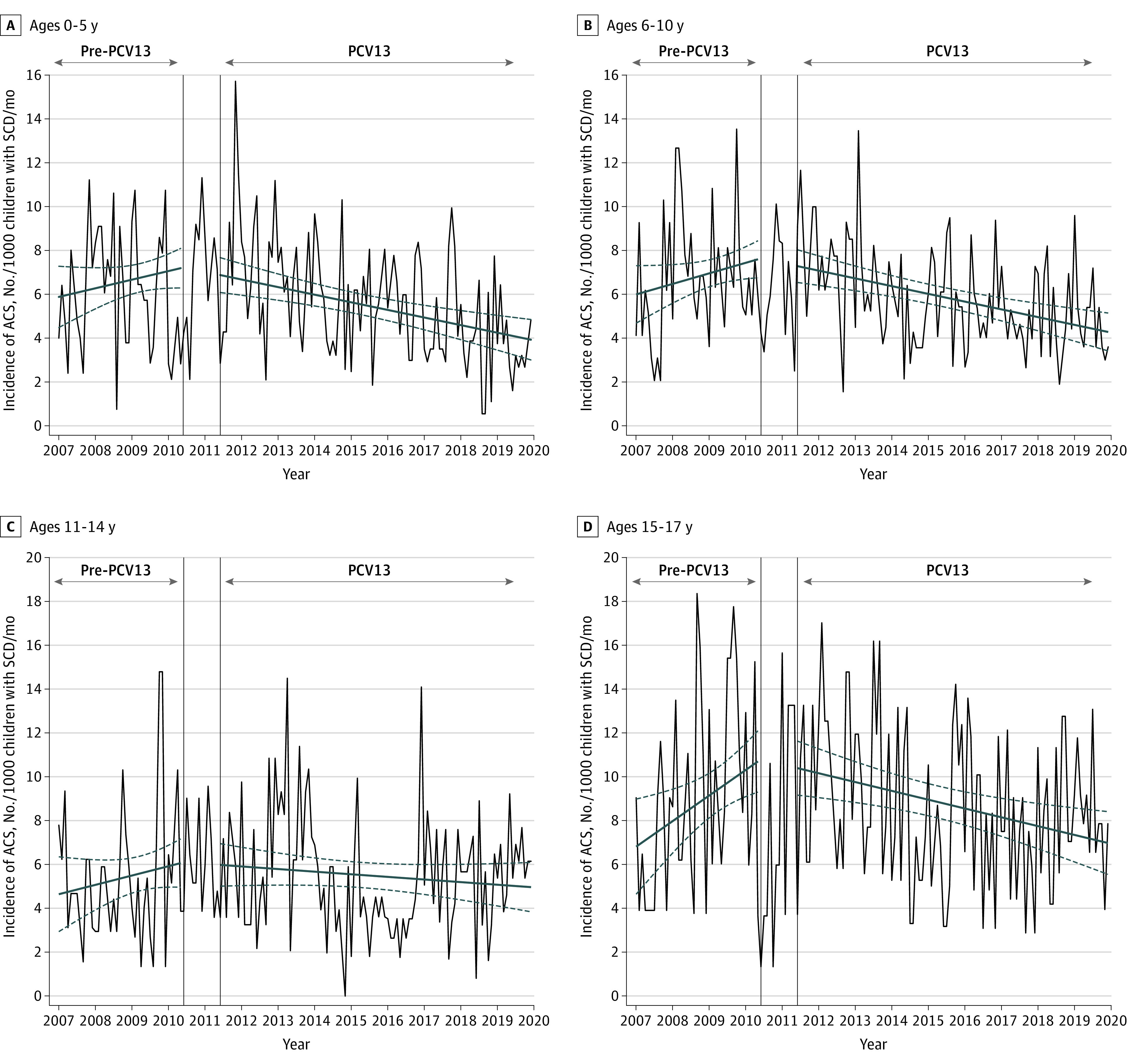
Association of 13-Valent Pneumococcal Conjugate Vaccine (PCV13) Implementation With the Monthly Incidence of Acute Chest Syndrome (ACS) per 1000 Children With Sickle Cell Disease (SCD) by Age Group The dark blue lines indicating the slope were estimated using the segmented linear regression model; dashed dark blue lines show the 95% CI. The vertical lines indicate the transition period during which PCV13 was implemented. The pre-PCV13 period was from January 2007 to May 2010; the transitional period, from June 2010 to May 2011; the PCV13 period, from June 2011 to December 2019.

The monthly incidence of ACS following PCV13 implementation was comparable for all age groups (cumulative decrease ranging from −34.3% for children aged 11 to 14 years to −52.8% for children aged 15 to 17 years) (Table 2; Figure 2). The proportion of ventilatory support and ICU transfer among patients with ACS did not significantly change following PCV13 implementation, suggesting that the severity of the disease remained unchanged (Table 2; eFigure 8 in the Supplement).

### Control Outcomes

**Figure 3.**
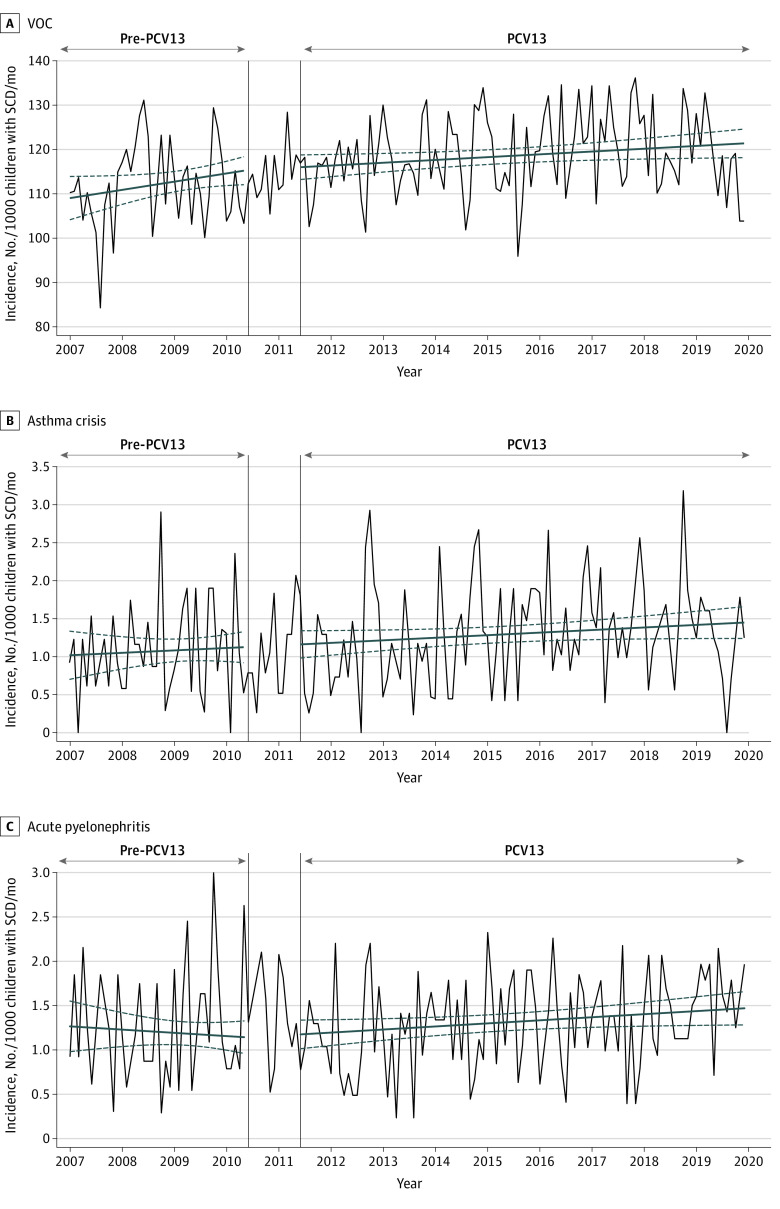
Evolution of the Monthly Incidence of Control Outcomes per 1000 Children With Sickle Cell Disease (SCD) The dark blue lines indicating the slope were estimated using the segmented linear regression model; dashed dark blue lines show the 95% CI. The vertical lines indicate the transition period during which 13-valent pneumococcal conjugate vaccine (PCV13) was implemented. The pre-PCV13 period was from January 2007 to May 2010; the transitional period, from June 2010 to May 2011; the PCV13 period, from June 2011 to December 2019. PCV indicates pneumococcal conjugate vaccine; VOC, vaso-occlusive crisis.

The incidence of VOC, asthma crises, and acute pyelonephritis per 1000 children with SCD did not significantly change over the study period (Table 2; Figure 3; eFigure 9 in the Supplement). This result in the control outcomes indicated that potential cointerventions were minimal and did not bias the association between the intervention and the decrease in ACS incidence.

## Discussion

PCV13 implementation in 2010 was associated with a marked decrease in the incidence of pediatric ACS in France, with a decline of 41.8% until December 2019. To our knowledge, this 13-year population-based ITS analysis is the first to provide data about the association of PCV13 implementation with reduced incidence of ACS.

The 40% decrease of ACS incidence following PCV13 implementation, suggesting an important pneumococcal involvement in this entity, contrasts with the 4.5% proportion of pneumococcal-related ACS suggested by Vichinsky et al.^4^ As for ACS, the precise role of *S pneumoniae* in community-acquired pneumonia has been difficult to estimate using standard microbiological methods. In this context, the vaccine probe approach has been proposed to address this issue. This epidemiological research method relies on estimating the population-level reduction of the disease following the implementation of a vaccine targeting the pathogen to elucidate its role in the disease. Using this approach, vaccine probe studies estimated the role of *S pneumoniae* to be as high as 40% in community-acquired pneumonia^12,26^ compared with 5% to 10% for microbiological studies.^27^ Several limitations in terms of the microbiological methods may explain their low capacity to accurately estimate the role of *S pneumoniae* in community-acquired pneumonia and in ACS. Molecular tests on nasopharyngeal sampling poorly differentiate pneumonia pathogens from upper airway colonizers. Furthermore, lower respiratory specimens can be contaminated by upper respiratory secretions and invasive pulmonary sampling is rarely performed.^28^ Although blood cultures are an important diagnostic tool for pneumonia, only 1% to 5% of patients admitted to the hospital with community-acquired pneumonia have documented bloodstream infections.^29^ Pneumococcal antigen detection tests in urine are still challenging in children because they often also detect pneumococcal carriage,^28^ even if promising tests are in development. Therefore, the vaccine probe approach appears to represent a reliable alternative method to estimate the etiological fraction of *S pneumoniae* in ACS.

Such a reduction in the incidence of ACS has not been reported following PPSV23 implementation in SCD populations nor PCV7 implementation. Several points merit discussion. The efficacy of PPSV23 has been demonstrated on IPD, but no effect on non-IPD, such as pulmonary involvement, has been found.^30^ Similarly, PCV7 implementation worldwide in the 2000s led to a major reduction in the incidence of IPD in the general population, including those with SCD,^7^ but its impact on the incidence of community-acquired pneumonia in children was moderate and transient and eroded by the emergence of serotypes 19A and 7F.^9,31^ By contrast, PCV13 offers strong protection against non-PCV7 serotypes 1, 3, 5, 7F, and 19A,^11,17^ which show pulmonary tropism and were the most frequently involved in documented community-acquired pneumonia in the pre-PCV13 era.^32^ This has been confirmed by numerous studies reporting a strong reduction in noninvasive pulmonary infections following PCV13 implementation.^26,33^ PCV13 would therefore be expected to significantly reduce ACS if *S pneumoniae* plays a role in this condition. Substantial serotype replacement has been recently reported in IPD in the general population and children with SCD.^34,35^ Several of these emerging non-PCV13 serotypes, such as serotype 8, show important pulmonary tropism.^36^ Two next-generation PCVs are currently being evaluated for approval and may cover these emerging non-PCV13 serotypes.^37,38^ Thus, the assessment of the potential public health benefit of such next-generation PCVs may include their potential to reduce ACS incidence in children with SCD.

By highlighting the important role of *S pneumoniae* in the onset of ACS, this study also raises questions regarding the therapeutic management of ACS. Bundy et al^19^ assessed the effectiveness of guideline-adherent antibiotic treatment (ie, macrolides and parenteral cephalosporin^39^) on hospital readmission. ACS treated with cephalosporins were associated with significantly lower 30-day (ACS-related and all-cause) readmission rates.^19^ Our study adds an indirect argument to support a specific anti-pneumococcal antibiotic therapy in ACS, which needs to be confirmed by further prospective comparative studies.^40^ Furthermore, the optimal age of discontinuation of daily oral prophylactic penicillin is still unclear, ranging from ages 5 to 15 years depending on the guidelines.^39,41^ Our exploratory subgroup analysis also showed a reduction in the incidence of ACS in older children following PCV13 implementation, which may require prospective comparative studies to assess the benefit of oral prophylaxis in such age groups.

### Limitations

Our study had several limitations. First, the diagnosis of ACS relies on nonspecific criteria that allow for possible clinical overlap with pneumonia. Indeed, although these 2 entities are theoretically distinct in the National Institutes of Health guidelines,^39^ they may be difficult to differentiate from a clinical point of view, and misclassification can occur. However, in this study, the characteristics of patients hospitalized for ACS were very different from those hospitalized for pneumonia in terms of median age, seasonal pattern, duration of stay, and proportion of ventilatory support and ICU transfer. Furthermore, ACS characteristics were similar to cases described in previous studies. A median age of 8.7 years was reported by Bundy et al^19^ compared with 8 years in our study. The median duration of stay of 7 days was consistent with the results of the study of Vichinsky et al (6.8 days),^42^ as was the proportion of ventilatory support and the seasonal variation in ACS incidence. In our study, the incidence of ACS of 7.3 per 1000 children with SCD before PCV13 implementation (ie, 8.8 per 100 person-years) was also in accordance with previous research.^2,43,44^ Furthermore, to account for potential misclassification between ACS and pneumonia, and as suggested by several previous studies,^19,20^ we conducted sensitivity analyses combining ACS and pneumonia and excluding the *ICD-10* code for pneumonia (J18.9) from ACS definition, all of which showed similar results.

Second, our analysis may have been affected by simultaneous cointerventions targeting the same outcome. Hydroxyurea treatment in children has allowed reduction in the rate of SCD-related acute clinical events, such as VOC and ACS.^45^ The French guidelines on SCD management were updated in 2010. Although the indication of hydroxyurea remained unchanged (for symptomatic forms of SCD in children aged over 2 years^41^), we cannot overlook the fact that the proportion of children treated with hydroxyurea increased following greater adherence to the updated guidelines. However, in our study, the sensitivity analysis that was adjusted for the incidence of VOC over time gave the same results. In addition, the incidence of VOC remained unchanged during the study period. These 2 arguments limit the possibility that changes observed in the incidence of ACS may have been related to changes in VOC incidence or hydroxyurea prescriptions. The national guidelines on prophylaxis with oral penicillin and early curative antibiotic treatment for febrile episodes did not change during the study period, nor did the guidelines regarding PPSV23 vaccination schedule,^18,46^ thus limiting the risk of bias. Finally, we cannot rule out that progressive improvement in the management of VOC, such as blood transfusion, pain management, incentive spirometry, and patient education^39^ also participated in reducing the incidence of ACS. However, no specific guidelines recommended changes in these preventive therapies during the study period.

Third, the identification of ACS admissions was based on *ICD-10* codes, and codification can evolve over time. Some specific codes for ACS provided in the National Center for Health Statistics *ICD-10-Clinical Modification*^47^ (ie, D57.01, D57.211, D57.411, D57.811) were not available in the PMSI database. However, the discharge diagnoses recorded in the PMSI undergo internal quality control (Medical Information Department^48^) and are subject to national recommendations from the ATIH that did not evolve over the study period. The unchanged proportions of *ICD-10* code combinations among patients with ACS—during the study period and between the age groups—suggest that our findings were not influenced by a revision of the PMSI coding system.

## Conclusions

PCV13 implementation in France was associated with a marked reduction in the incidence of ACS among children with SCD. These findings provide new evidence for the underestimated involvement of *S pneumoniae* in childhood ACS. The assessment of the potential public health benefit of next-generation PCVs should include their potential to reduce ACS incidence among children with SCD.

## Notes

Supplement. **eTable 1.** Clinical Diagnoses Defined by *ICD-10* Code Combinations **eFigure 1.** Number of Children <18 Years of Age With SCD Living in France Over Time **eFigure 2.** Number of Children With SCD Living in France Over Time, by Age Group **eTable 2.** Proportions of *ICD-10* Code Combinations Among ACS Cases Over Time and by Age Group **eTable 3.** Preintervention and Postintervention Time Trends for the Monthly Incidence of ACS per 1000 Children With SCD (January 2007 to December 2019) **eFigure 3.** Correlograms and Residuals Analysis of the Final Segmented Regression Model for the Monthly Incidence of ACS per 1000 Children With SCD **eFigure 4.** Association of PCV13 Implementation With the Monthly Incidence of Combined ACS and Pneumonia per 1000 Children With SCD in France (N = 5796) **eFigure 5.** Correlograms and Residuals Analysis of the Segmented Regression Model for the Monthly Incidence of Combined ACS and Pneumonia per 1000 Children With SCD **eTable 4.** Association of PCV13 Implementation With the Monthly Incidence of Pneumonia per 1000 Children With SCD in France (N = 1789) **eFigure 6.** Correlograms and Residuals Analysis of the Segmented Regression Model for the Monthly Incidence of Pneumonia per 1000 Children With SCD **eFigure 7.** Correlograms and Residuals Analysis of the Sensitivity Analyses **eFigure 8.** Correlograms and Residuals Analysis of Secondary Analyses **eFigure 9.** Correlograms and Residuals Analysis of Control Outcomes
