# Supplementary material for: Incidence of Acute Chest Syndrome in Children With Sickle Cell Disease Following Implementation of the 13-Valent Pneumococcal Conjugate Vaccine in France
Source: JAMA Netw Open. 2022 Aug 2;5(8):e2225141. doi: 10.1001/jamanetworkopen.2022.25141 (PMC9346553; doi:10.1001/jamanetworkopen.2022.25141)
Supplement: Supplement. — eTable 1. Clinical Diagnoses Defined by ICD-10 Code Combinations eFigure 1. Number of Children <18 Years of Age With SCD Living in France Over Time eFigure 2. Number of Children With SCD Living in France Over Time, by Age Group eTable 2. Proportions of ICD-10 Code Combinations Among ACS Cases Over Time and by Age Group eTable 3. Preintervention and Postintervention Time Trends for the Monthly Incidence of ACS per 1000 Children With SCD (January 2007 to December 2019) eFigure 3. Correlograms and Residuals Analysis of the Final Segmented Regression Model for the Monthly Incidence of ACS per 1000 Children With SCD eFigure 4. Association of PCV13 Implementation With the Monthly Incidence of Combined ACS and Pneumonia per 1000 Children With SCD in France (N = 5796) eFigure 5. Correlograms and Residuals Analysis of the Segmented Regression Model for the Monthly Incidence of Combined ACS and Pneumonia per 1000 Children With SCD eTable 4. Association of PCV13 Implementation With the Monthly Incidence of Pneumonia per 1000 Children With SCD in France (N = 1789) eFigure 6. Correlograms and Residuals Analysis of the Segmented Regression Model for the Monthly Incidence of Pneumonia per 1000 Children With SCD eFigure 7. Correlograms and Residuals Analysis of the Sensitivity Analyses eFigure 8. Correlograms and Residuals Analysis of Secondary Analyses eFigure 9. Correlograms and Residuals Analysis of Control Outcomes [file jamanetwopen-e2225141-s001.pdf]

Supplemental Online Content

Assad Z, Michel M, Valtuille Z, et al. Incidence of acute chest syndrome in children with sickle cell disease following implementation of the 13-valent pneumococcal conjugate vaccine in France. *JAMA Netw Open.* 2022;5(8):e2225141. doi:10.1001/jamanetworkopen.2022.25141

eTable 1. Clinical Diagnoses Defined by *ICD-10* Code Combinations

eFigure 1. Number of Children <18 Years of Age With SCD Living in France Over Time

eFigure 2. Number of Children With SCD Living in France Over Time, by Age Group

eTable 2. Proportions of *ICD-10* Code Combinations Among ACS Cases Over Time and by Age Group

eTable 3. Preintervention and Postintervention Time Trends for the Monthly Incidence of ACS per 1000 Children With SCD (January 2007 to December 2019)

eFigure 3. Correlograms and Residuals Analysis of the Final Segmented Regression Model for the Monthly Incidence of ACS per 1000 Children With SCD

eFigure 4. Association of PCV13 Implementation With the Monthly Incidence of Combined ACS and Pneumonia per 1000 Children With SCD in France (N = 5796)

eFigure 5. Correlograms and Residuals Analysis of the Segmented Regression Model for the Monthly Incidence of Combined ACS and Pneumonia per 1000 Children With SCD

eTable 4. Association of PCV13 Implementation With the Monthly Incidence of Pneumonia per 1000 Children With SCD in France (N = 1789)

eFigure 6. Correlograms and Residuals Analysis of the Segmented Regression Model for the Monthly Incidence of Pneumonia per 1000 Children With SCD

eFigure 7. Correlograms and Residuals Analysis of the Sensitivity Analyses

eFigure 8. Correlograms and Residuals Analysis of Secondary Analyses

eFigure 9. Correlograms and Residuals Analysis of Control Outcomes

This supplemental material has been provided by the authors to give readers additional information about their work.

© 2022 Assad Z et al. *JAMA Network Open.*

eTable 1. Clinical Diagnoses Defined by *ICD-10* Code Combinations

Diagnosis	ICD-10 code combinations		
Acute chest syndrome	D570 (Hb-SS disease with crisis)<br>or D572 (Sickle-cell/Hb-C disease)	and	J960 (acute respiratory failure)<br>or J189 (pneumonia, unspecified organism)<br>or I269 (pulmonary embolism without acute pulmonary heart disease)
Asthma crisis with SCD	D571 (sickle-cell disease without crisis)<br>or D578 (other sickle cell disorders)	and	J45 (asthma)<br>or J450 (allergic asthma)<br>or J451 (non-allergic asthma)<br>or J458 (associated asthma)<br>or J459 (other and unspecified asthma)<br>or J46 (severe asthma)
Pneumonia with SCD	D571 or D578	and	J13 (pneumonia due to Streptococcus pneumoniae)<br>or J159 (unspecified bacterial pneumonia)<br>or J18 (pneumonia, unspecified organism)<br>or J180 (bronchopneumonia, unspecified organism)<br>or J181(lobar pneumonia, unspecified organism)<br>or J189 (pneumonia, unspecified organism)<br>or J851 (abscess of lung with pneumonia)<br>or J90 (pleural effusion, not elsewhere classified)
Other LRTI with SCD	D571 or D578	and	J101 (influenza due to other identified influenza virus with other respiratory manifestations)<br>or J111 (influenza due to unidentified influenza virus with other respiratory manifestations)<br>or J20 (acute bronchitis)<br>or J209 (acute bronchitis, unspecified)<br>or J21 (acute bronchiolitis)<br>or J210 (acute bronchiolitis due to respiratory syncytial virus)<br>or J211 (acute bronchiolitis due to human Metapneumovirus)<br>or J218 (acute bronchiolitis due to other specified organisms)<br>or J219 (acute bronchiolitis, unspecified)<br>or J22 (unspecified acute lower respiratory infection)
Acute pyelonephritis with SCD	D570 or D571 or D572 or D578	and	N10 (acute pyelonephritis)<br>or N136 (pyonephrosis)
Vaso-occlusive crisis	D570 or D572	and	no diagnosis for other acute SCD

Hb: hemoglobin, ICD: International Classification of Diseases, LRTI: lower respiratory-tract infections, SCD: sickle-cell disease.

eFigure 1. Number of Children <18 Years of Age With SCD Living in France Over Time

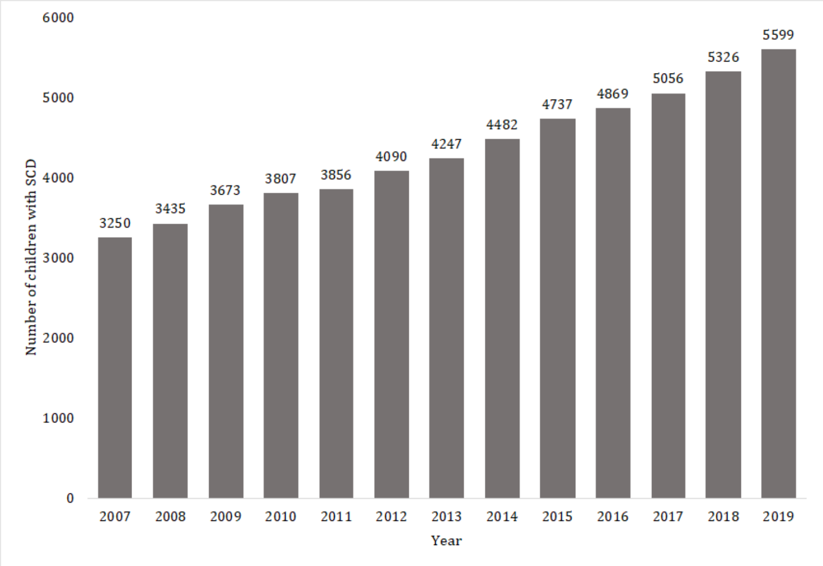

Data provided by the National Health Insurance Scheme database (https://assurance-maladie.ameli.fr/etudes-et-donnees/prevalence-beneficiaires-ald-2019).
SCD: sickle-cell disease

© 2022 Assad Z et al. *JAMA Network Open.*

eFigure 2. Number of Children With SCD Living in France Over Time, by Age Group

a) **children aged 0-5 years**
b) **children aged 6-10 years**
c) **children aged 11-14 years**
d) **children aged 15-17 years**

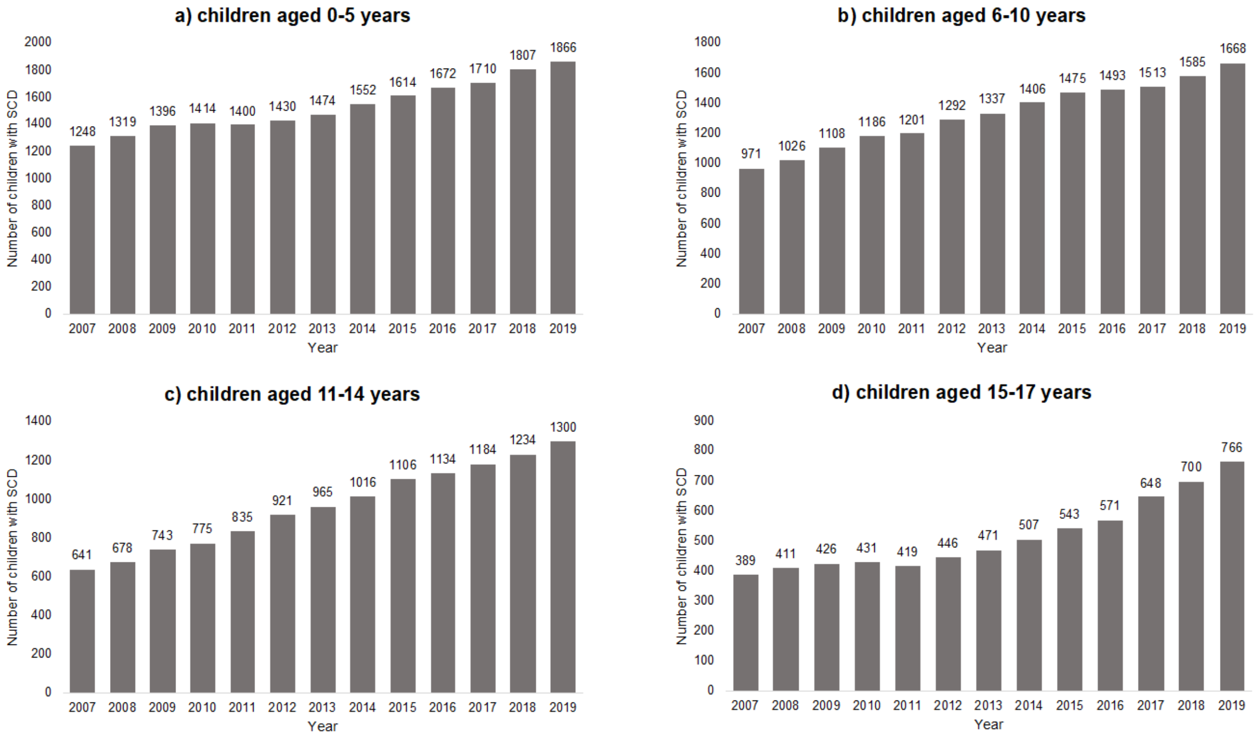

Data provided by the National Health Insurance Scheme database (https://assurance-maladie.ameli.fr/etudes-et-donnees/prevalence-beneficiaires-ald-2019).
SCD: sickle-cell disease.

© 2022 Assad Z et al. *JAMA Network Open.*

eTable 2. Proportions of *ICD-10* Code Combinations Among ACS Cases Over Time and by Age Group

	D570 and J960	D570 and J189	D570 and I269	**Total D570**	D572 and J960	D572 and J189	D572 and I269	**Total D572**	**Total ACS cases**
**Number of cases**	2,556 (63.8)	1,931 (48.2)	94 (2.3)	3,938 (98.3)	43 (1.1)	55 (1.4)	5 (0.1)	92 (2.3)	4,007
**By period**									
Pre PCV13 period	537 (58.7)	467 (51.0)	16 (1.7)	895 (97.8)	11 (1.2)	21 (2.3)	2 (0.2)	29 (3.2)	915
PCV 13 period	1,836 (65.7)	1,327 (47.5)	66 (2.4)	2,754 (98.5)	25 (0.9)	29 (1.0)	3 (0.1)	53 (1.9)	2,795
**By age group**									
0-5 years old	746 (54.6)	743 (54.4)	30 (2.2)	1,333 (97.5)	19 (1.4)	30 (2.2)	0	41 (3.0)	1,367
6-10 years old	825 (66.8)	601 (48.7)	16 (1.3)	1,216 (98.5)	11 (0.9)	16 (1.3)	2 (0.2)	26 (2.1)	1,235
11-14 years old	582 (70.7)	354 (43.0)	23 (2.8)	817 (99.3)	7 (0.9)	3 (0.4)	2 (0.2)	12 (1.5)	823
15-17 years old	402 (69.2)	233 (40.1)	25 (4.3)	571 (98.3)	6 (1.0)	6 (1.0)	1 (0.2)	13 (2.2)	581
Missing	1	0	0	0	0	0	0	0	1

Data are presented as numbers (%).
ACS: acute chest syndrome, ICD-10: International Classification of Diseases, Tenth Revision, PCV: pneumococcal conjugate vaccine.

© 2022 Assad Z et al. *JAMA Network Open.*

eTable 3. Preintervention and Postintervention Time Trends for the Monthly Incidence of ACS per 1000 Children With SCD (January 2007 to December 2019)

	Time trend, pre-intervention period			Time trend, post-intervention period		
	Estimate	Standard error	P value	Estimate	Standard error	P value for slope change
Segmented linear regression model with transition period (main analysis)	0.04	0.02	.01	-0.07	0.02	<.001
Quasi-Poisson regression model	0.005	0.002	.01	-0.009	0.003	<.001
Segmented linear regression model with trigonometric function (12 m)	0.03	0.01	.01	-0.06	0.02	<.001
Segmented linear regression model with trigonometric function (3-6-12 m)	0.03	0.01	.01	-0.06	0.02	<.001
Model adjusted for the monthly incidence of VOC	0.04	0.02	.03	-0.06	0.02	.001
Model with combined ACS and pneumonia	0.03	0.02	.14	-0.05	0.02	.03
Model with J189 and D572 excluded from ACS definition	0.05	0.01	<.001	-0.07	0.02	<.001
Segmented linear regression model excluding transition period	0.03	0.01	.02	-0.06	0.02	.002

Pre- intervention time trend is presented as the monthly percentage change from ACS incidence in January 2007.
Post-intervention time trend is presented as the monthly percentage change from ACS incidence in May 2010.
ACS: acute chest syndrome, SCD: sickle-cell disease, VOC: vaso-occlusive crisis, J189: ICD-10 code for pneumonia, D572: ICD-10 code for sickle-cell/Hemoglobin-C disease.

© 2022 Assad Z et al. *JAMA Network Open.*

eFigure 3. Correlograms and Residuals Analysis of the Final Segmented Regression Model for the Monthly Incidence of ACS per 1000 Children With SCD

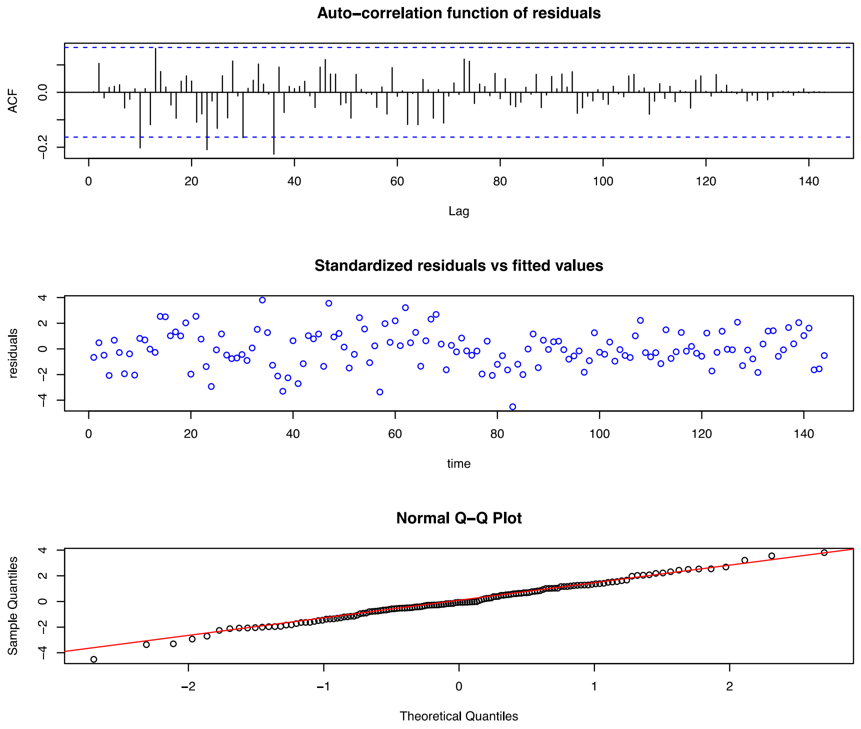

ACF: autocorrelation function.

© 2022 Assad Z et al. *JAMA Network Open.*

eFigure 4. Association of PCV13 Implementation With the Monthly Incidence of Combined ACS and Pneumonia per 1000 Children With SCD in France (N = 5796)

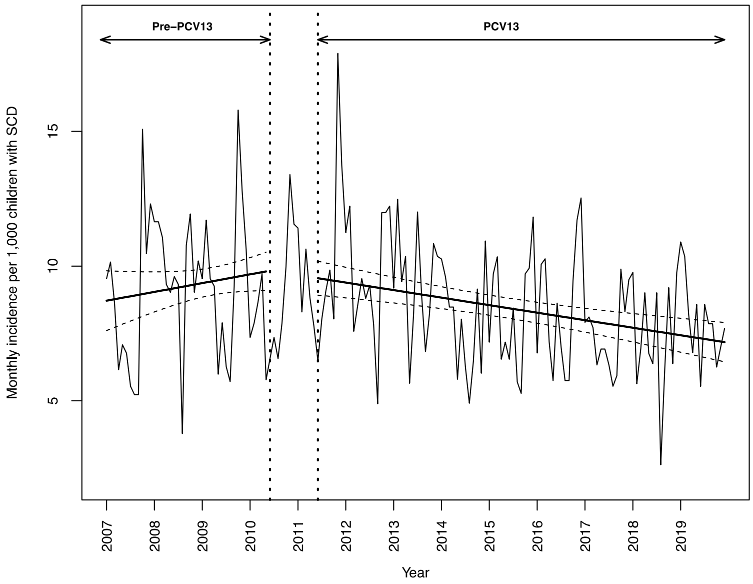

The bold lines for the slope were estimated using the segmented linear regression model. The dotted lines show the 95% CI. The dotted vertical lines indicate the transition period during which PCV13 was implemented. Pre-PCV13: period from January 2007 to May 2010. Transitional period: from June 2010 to May 2011. PCV13: period from June 2011 to December 2019. ACS: acute chest syndrome, SCD: sickle-cell disease, PCV: pneumococcal conjugate vaccine, CI: confidence interval.

© 2022 Assad Z et al. *JAMA Network Open.*

eFigure 5. Correlograms and Residuals Analysis of the Segmented Regression Model for the Monthly Incidence of Combined ACS and Pneumonia per 1000 Children With SCD

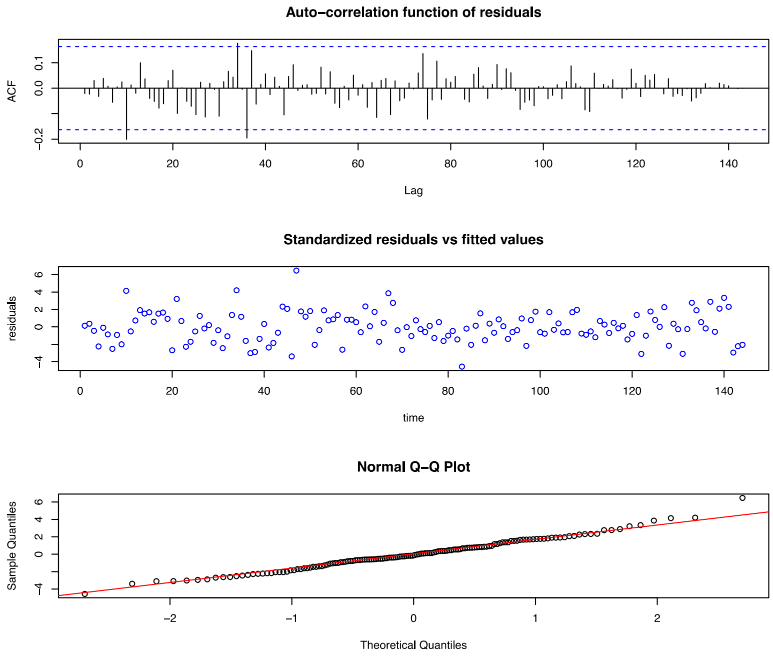

ACF: autocorrelation function.

© 2022 Assad Z et al. *JAMA Network Open.*

eTable 4. Association of PCV13 Implementation With the Monthly Incidence of Pneumonia per 1000 Children With SCD in France (N = 1789)

	Change in slope per month % [95 CI]	P Value for slope change	Estimated cumulative change by the end of the study % [95% CI]
Monthly incidence of pneumonia per 1,000 children with SCD[¥$]	-0.4 [-1.1; 0.2]	.15	-22.0 [-59.2; 15.2]

Data are presented as the monthly percentage change and total cumulative change (95% CI). All percentage changes are expressed as absolute change in percentage per month.

[¥] Monthly incidence expressed as the number of cases per 1,000 children with SCD.

[$] Analysis by segmented linear regression.

ACS: acute chest syndrome, CI=confidence interval, SCD: sickle-cell disease.

© 2022 Assad Z et al. *JAMA Network Open.*

eFigure 6. Correlograms and Residuals Analysis of the Segmented Regression Model for the Monthly Incidence of Pneumonia per 1000 Children With SCD

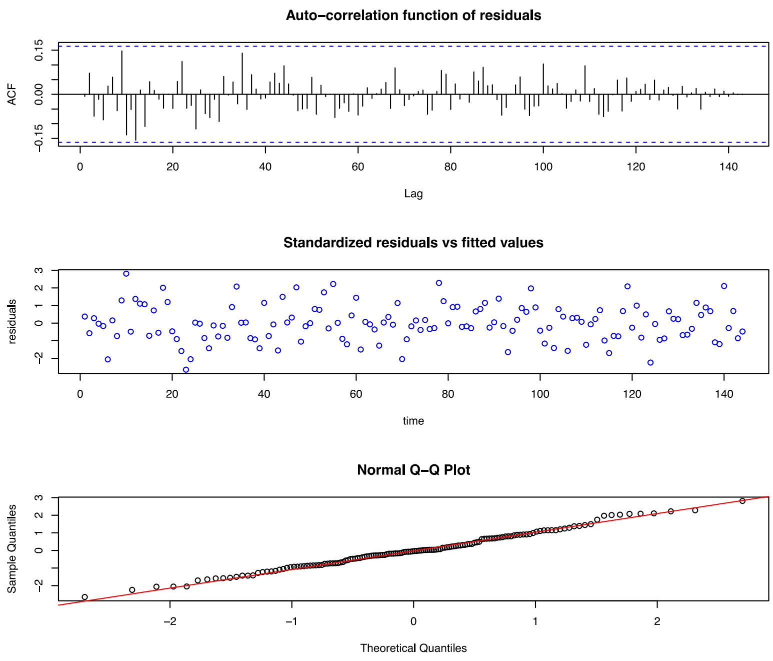

ACF: autocorrelation function.

© 2022 Assad Z et al. *JAMA Network Open.*

eFigure 7. Correlograms and Residuals Analysis of the Sensitivity Analyses

a) Quasi-Poisson regression model

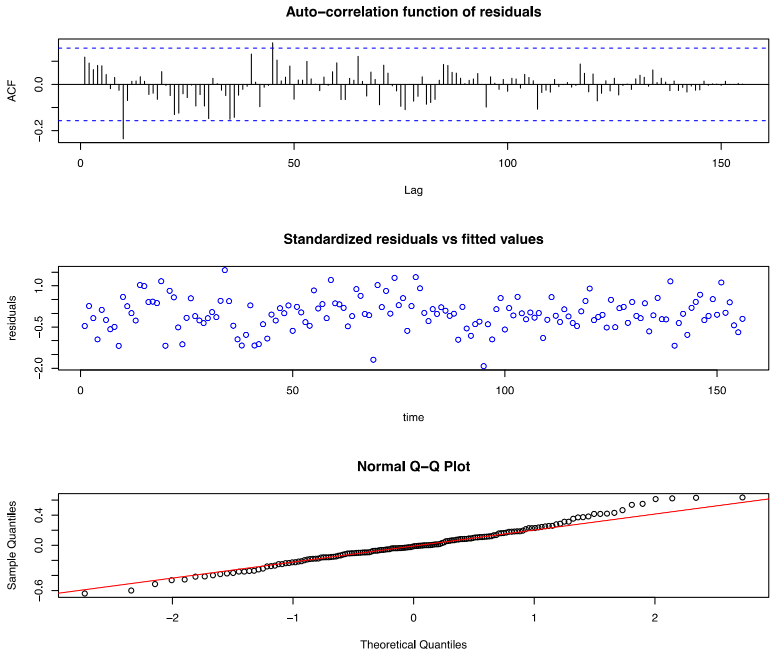

ACF: autocorrelation function.

b) Segmented linear regression model with trigonometric function (12-month period)

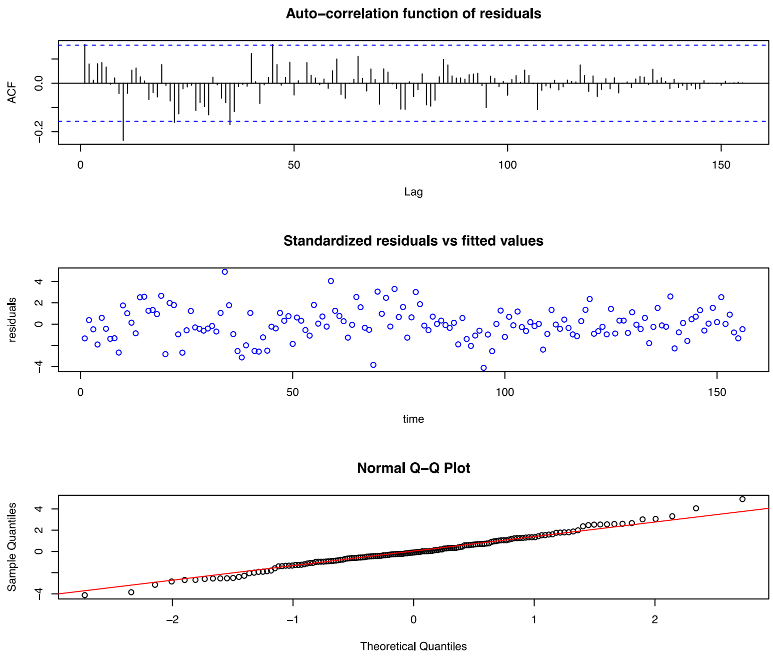

ACF: autocorrelation function.

© 2022 Assad Z et al. *JAMA Network Open.*

c) Segmented linear regression model with trigonometric function (3-6-12-month periods)

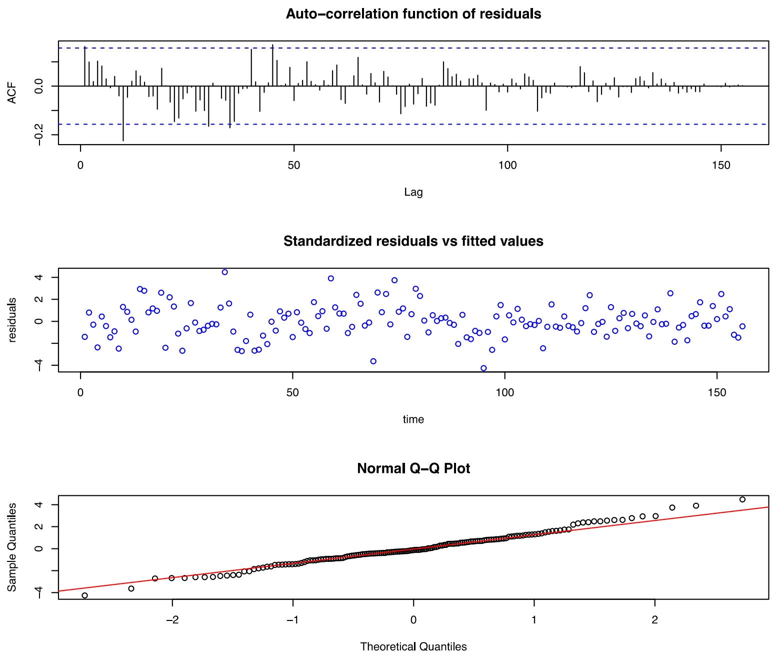

ACF: autocorrelation function.

d) Segmented linear regression model without transitional period

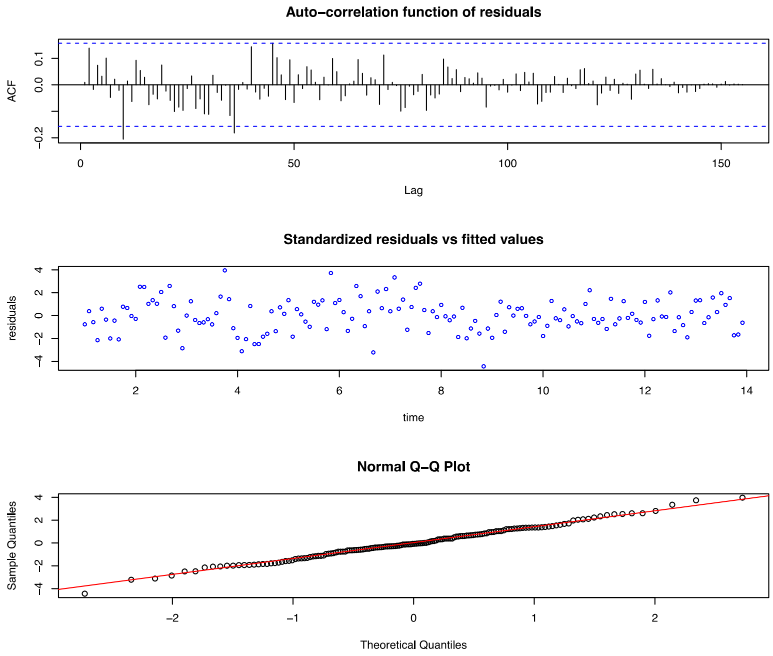

ACF: autocorrelation function.

© 2022 Assad Z et al. *JAMA Network Open.*

e) Segmented linear regression adjusted for the monthly incidence of VOC over the same period

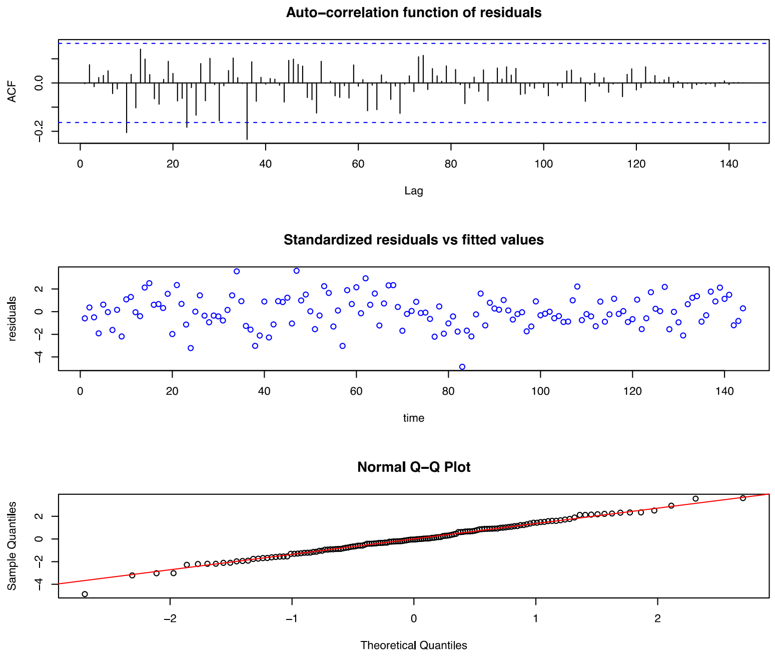

ACF: autocorrelation function.

f) Segmented linear regression model with J189 (pneumonia, unspecified organism) and D572 (sickle-cell/Hemoglobin-C disease) codes excluded from ACS definition

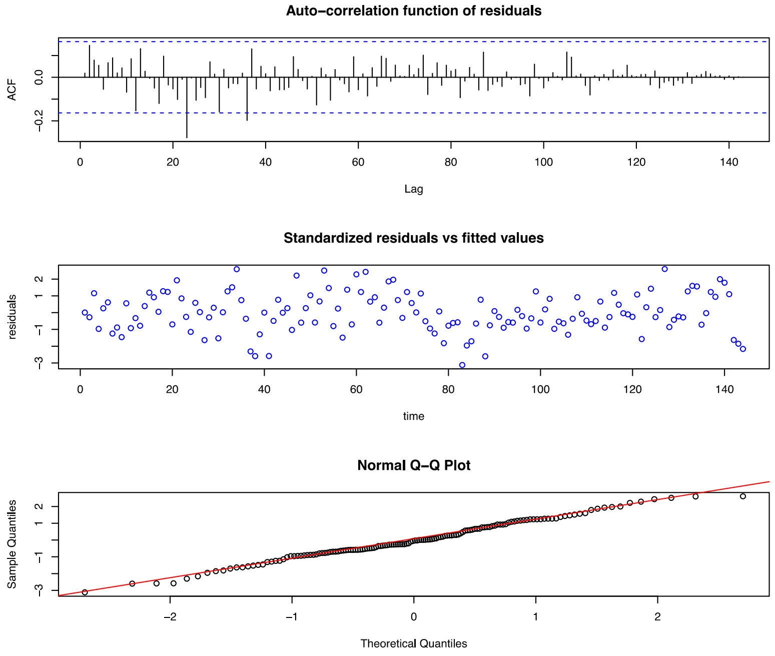

ACF: autocorrelation function.

© 2022 Assad Z et al. *JAMA Network Open.*

eFigure 8. Correlograms and Residuals Analysis of Secondary Analyses

a) Segmented regression model for the monthly incidence of ACS per 1,000 children with SCD aged 0-5 years

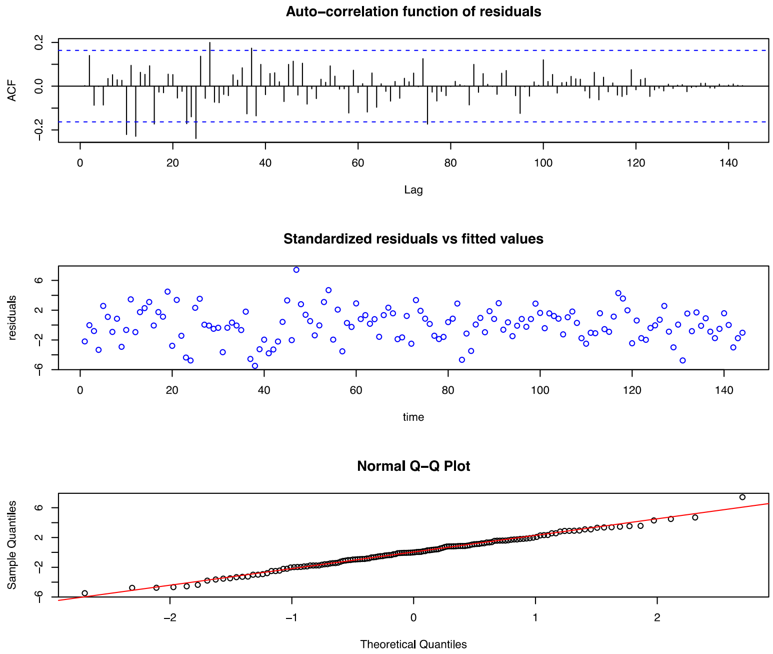

ACF: autocorrelation function.

b) Segmented regression model for the monthly incidence of ACS per 1,000 children with SCD aged 6-10 years

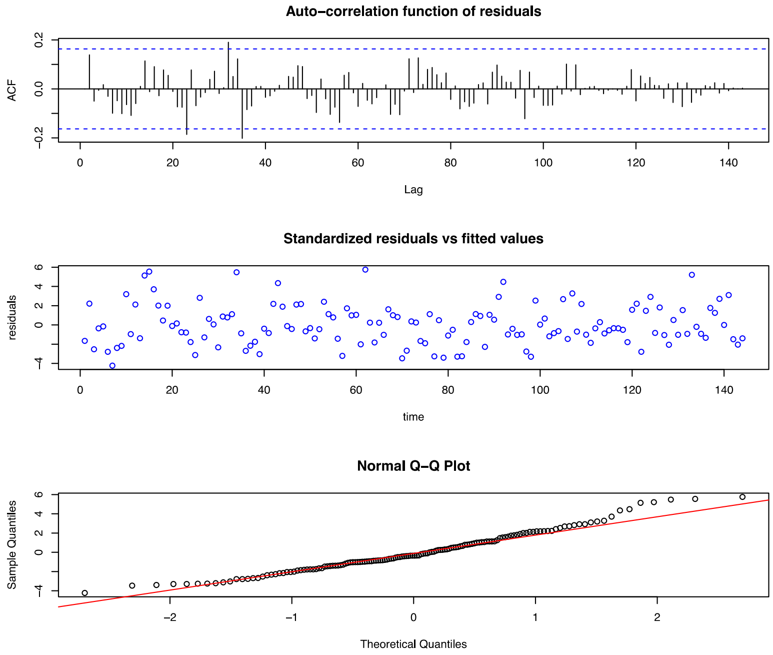

ACF: autocorrelation function.

© 2022 Assad Z et al. *JAMA Network Open.*

c) Segmented regression model for the monthly incidence of ACS per 1,000 children with SCD aged 11-14 years

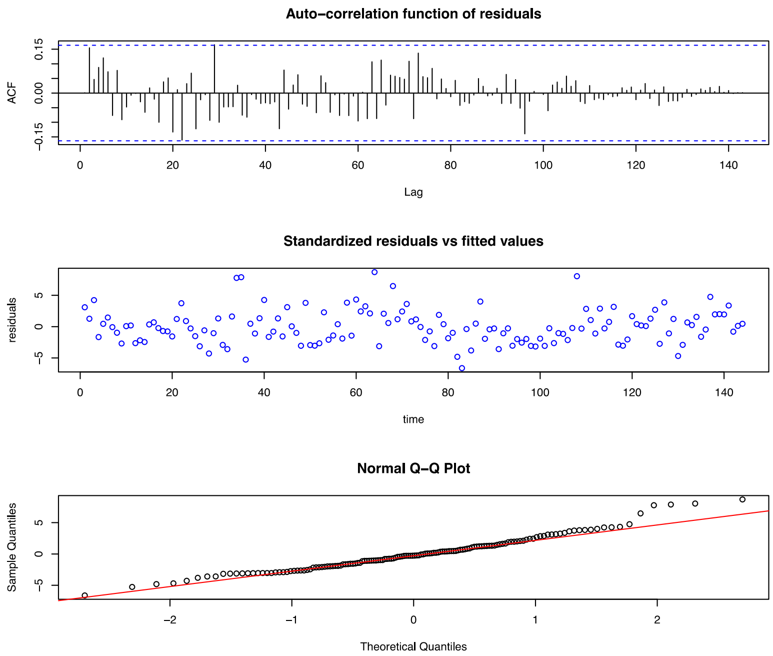

ACF: autocorrelation function.

d) Segmented regression model for the monthly incidence of ACS per 1,000 children with SCD aged 15-17 years

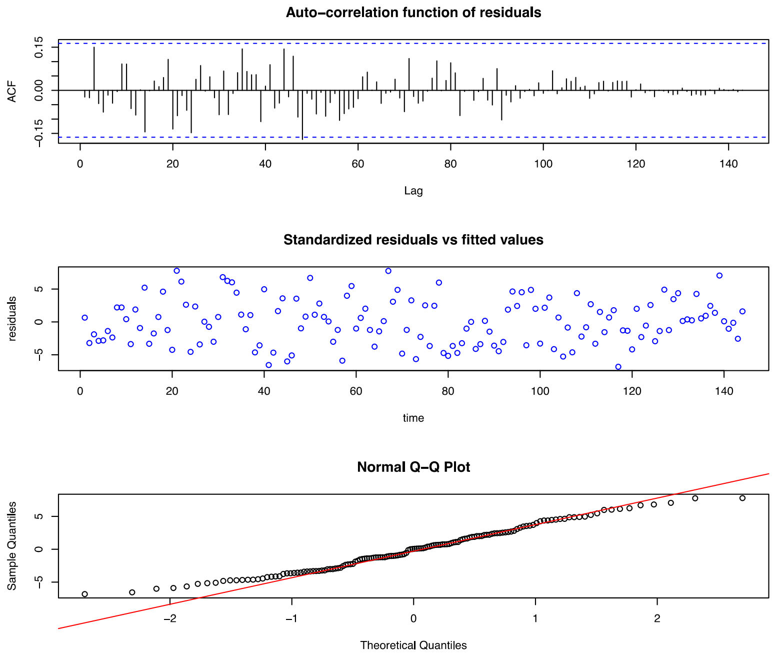

ACF: autocorrelation function.

© 2022 Assad Z et al. *JAMA Network Open.*

e) Segmented regression model for the proportion of ventilatory support among ACS episodes over time

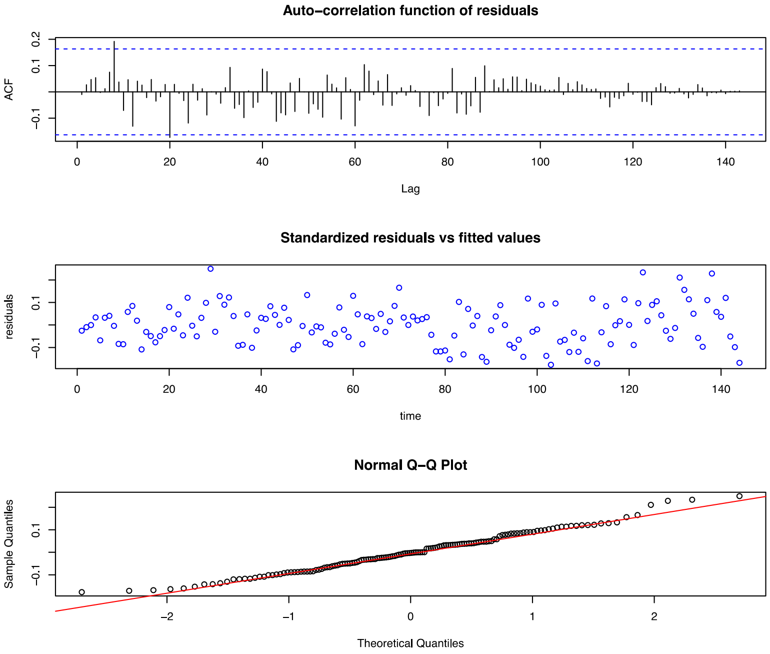

ACF: autocorrelation function.

f) Segmented regression model for the proportion of transfer to intensive care unit (ICU) among ACS episodes over time

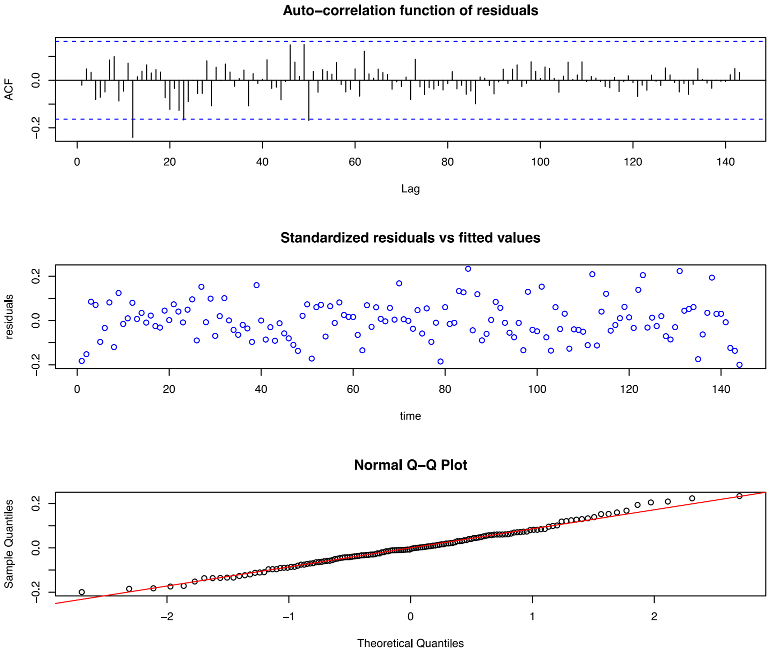

ACF: autocorrelation function.

© 2022 Assad Z et al. *JAMA Network Open.*

eFigure 9. Correlograms and Residuals Analysis of Control Outcomes

a) Segmented regression model for the monthly incidence of VOC per 1,000 children with SCD

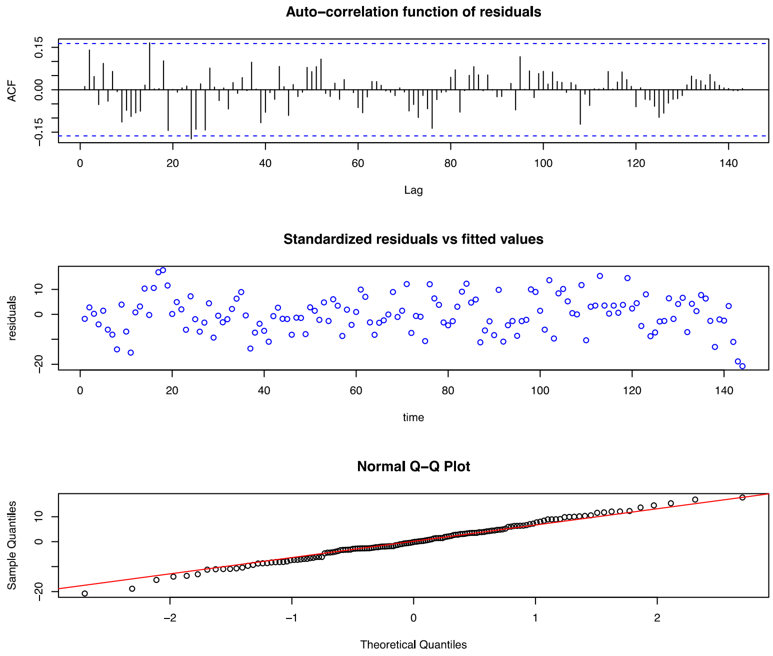

ACF: autocorrelation function.

b) Segmented regression model for the monthly incidence of asthma crisis per 1,000 children with SCD

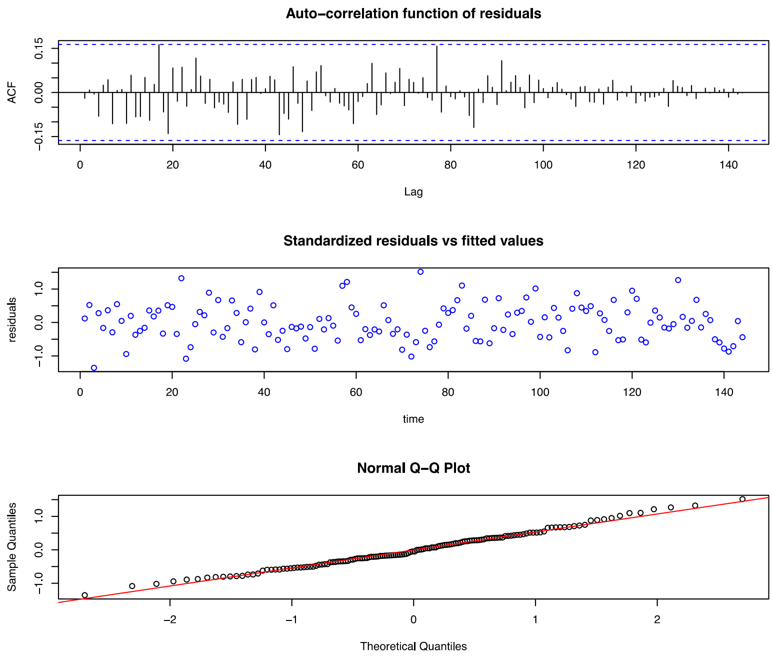

ACF: autocorrelation function.

© 2022 Assad Z et al. *JAMA Network Open.*

c) Segmented regression model for the monthly incidence of acute pyelonephritis per 1,000 children with SCD

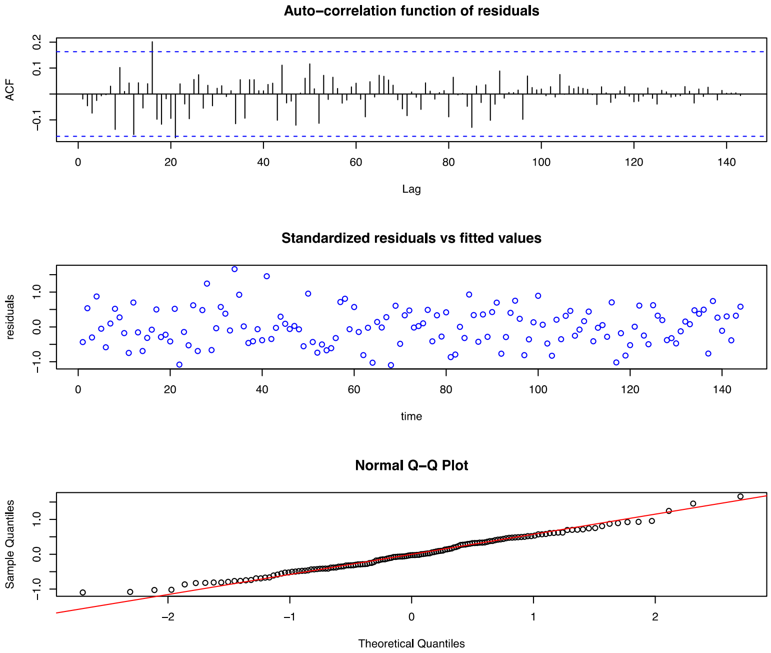

ACF: autocorrelation function.

© 2022 Assad Z et al. *JAMA Network Open.*
